# Estimation of the distribution function of a finite population utilizing auxiliary information in the context of non-response within complex survey sampling

**DOI:** 10.1371/journal.pone.0322660

**Published:** 2025-05-22

**Authors:** Mohsin Abbas, Muhammad Ahmed Shehzad, Haris Khurram, Mahwish Rabia

**Affiliations:** 1 Department of Statistics, Bahauddin Zakariya University, Multan, Pakistan; 2 Department of Science and Humanities, National University of Computer and Emerging Sciences, Chiniot - Faisalabad Campus, Pakistan; 3 Department of Statistics, Government College Women University Sialkot, Pakistan; RMIT University, AUSTRALIA

## Abstract

This study focuses on estimating a finite population cumulative distribution function (CDF) using two-stage and three-stage cluster sampling under non-response. This work is then extended to estimate the finite population CDF under non-response using stratified two-stage and three-stage cluster sampling. We propose two distinct families of CDF estimators, specifically designed for these complex surveys, namely classical ratio/product-type and exponential ratio/product-type. Furthermore, we introduce a difference estimator for the CDF under non-response, utilizing ancillary information about the variances and covariances of the estimators under these complex schemes. We provide mathematical expressions for the biases and mean squared errors of the proposed CDF estimators, based on first-order approximation. To evaluate the performance of the proposed estimators, we conduct extensive simulations and assess their efficiency. The simulation results demonstrate that the proposed families of estimators perform well under different sampling scenarios. Our findings indicate that difference CDF estimators are more explicit than the other estimators discussed. We support our theoretical claims by analyzing real datasets.

## 1 Introduction

Non-response is a critical issue in survey sampling that can lead to biased estimates and inefficiencies in statistical inference. In complex survey sampling schemes such as two-stage cluster sampling (2SCS), stratified two-stage cluster sampling (S2SCS), three-stage cluster sampling (3SCS), and stratified three-stage cluster sampling (S3SCS), the challenge of non-response becomes even more pronounced due to the hierarchical structure of the data and the varying probabilities of response at different stages. Despite the extensive literature on CDF estimation, existing methods primarily focus on simple random sampling (SRS) or basic stratified sampling, with limited attention to the impact of non-response in complex sampling frameworks.

A significant gap in the literature is the absence of comprehensive methods for estimating the finite population CDF under non-response within these complex sampling schemes while effectively incorporating auxiliary information. Traditional estimation approaches often neglect the potential of utilizing ancillary information such as variances and covariances of estimators, which could significantly enhance estimation efficiency. Moreover, the use of ratio/product-type and exponential ratio/product-type estimators in handling non-response within complex sampling designs remains under-explored.

It is common for surveys to have incomplete data due to certain reasons. For example, in an opinion survey, the selected family may have moved to another place or the selected person may have passed away. In a mailed questionnaire, many respondents may not reply. This problem of incomplete sample data due to the non-availability of information from the respondent is called the problem of non-response. The estimate attained from such deficient data can be inaccurate, especially when there are differences between the respondents and non-respondents. One way to address the problem of non-response in a mailed questionnaire survey is to divide the population into two groups: respondents and non-respondents. From the non-respondent group, a sub-sample is selected and personally interviewed to obtain the necessary information. This technique is known as sub-sampling and was introduced by [7]. By making this extra effort, an unbiased estimator can be obtained for the non-response cases.

Estimating the CDF for a finite population is a fundamental problem in survey sampling, particularly in scenarios where auxiliary information is available. Accurate estimation of the CDF is crucial in various fields, including public health, economics, and social sciences, where decision-making often relies on understanding the distribution of a population’s characteristics. However, the complexity of survey designs, such as multi-stage cluster sampling with or without stratification, coupled with the challenge of non-response, complicates the estimation process. Non-response, a common issue in surveys, can introduce significant bias, leading to inaccurate estimates if not properly accounted for.

The estimation of the CDF in finite populations has been extensively studied in the context of survey sampling. Early works by [4] and [22] laid the foundation for the use of supplementary information in improving the exactness of survey estimates. The ratio and product estimators, introduced by these pioneers, have been particularly popular due to their ability to reduce the variance of estimators by leveraging the correlation between the study variable and auxiliary information. These estimators have since been widely applied in various sampling schemes, including SRS and stratified sampling, see [2,6,11–13] for more details.

In 2SCS, the population is divided into primary sampling units (PSUs), a subset of of the PSUs is then selected, and secondary sampling units (SSUs) are drawn form the selected PSUs. 3SCS adds an additional level by selecting tertiary sampling units (TSUs) from within subgroups of the chosen SSUs. For more details, see [4,8,10] and the references cited therein. S2SCS and S3SCS further improve the precision by dividing thr population into distinct strata based on certain characteristics before applying the sampling stages. This ensure better representation of subgroups, reduce variability, and improves the accuracy of estimates, particularly when the population is heterogeneous. These methods are particularly effective for large, dispersed, and diverse populations. For more details about these sampling schemes, the reader may see [1,8,15,17–19,21,22,24,25,30] for more details.

With the increasing complexity of survey designs, particularly in multi-stage sampling, researchers have sought to adapt these classical estimators to more complex scenarios. [8] provided early treatments of multi-stage sampling, highlighting the challenges and potential solutions for variance estimation. Following this, several authors, including [1,3,5,13,19,26,29,32], extended ratio and product estimators to multi-stage sampling frameworks, demonstrating their effectiveness in reducing bias and variance.

The issue of non-response has been a persistent challenge in survey sampling, leading to biased estimates if not properly addressed. [16] provided a comprehensive treatment of methods to handle non-response, including imputation techniques and weighting adjustments. In the context of CDF estimation, [23] proposed methods for handling non-response, but these approaches often involve complex adjustments that may not be feasible in all survey contexts.

Recent advancements have seen the development of exponential ratio and product estimators, which have been shown to offer improved performance in certain situations. These estimators, discussed by [29], are particularly useful when the relationship between the study variable and auxiliary information is non-linear. However, despite these innovations, the need for more precise estimators, particularly in complex sampling designs with non-response, remains.

To address this gap, this study develops novel estimation strategies for CDF estimation under non-response in 2SCS/S2SCS, 3SCS/S3SCS schemes. By incorporating auxiliary information, we propose two new families of CDF estimators: classical ratio/product-type and exponential ratio/product-type estimators. Additionally, we introduce a difference estimator specifically designed to enhance estimation accuracy. We derive mathematical expressions for the biases and mean squared errors of these estimators using first-order approximation and validate their performance with real-world datasets. Our findings indicate that the difference CDF estimators provide greater precision compared to the other proposed estimators, making them a more reliable approach for handling non-response in complex survey designs.

The paper is structured as follows: [section:2]Section 2 offers a concise overview of CDF estimation using the SRS scheme under non-response. Section 3 extends the discussion to CDF estimation under 2SCS/S2SCS and 3SCS/S3SCS schemes, also considering non-response. In Section 4 we derive precise mathematical formulations for the covariances associated with the CDF estimators within the 2SCS/S2SCS and 3SCS/S3SCS frameworks. Section 5 introduces two-families of estimators—ratio/product and exponential ratio/product—designed to estimate the population CDF under non-response conditions. An empirical analysis is presented in Section 6. Finally, Section 7 provides a summary of the key findings and concludes the paper.

## 2 Estimation of finite population CDF under non-response

Consider the scenario where we categorize the target population into two uniform groups, referred to as strata: (i) the response group; and (ii) the non-response group. Let $M^{\prime }$ denote the number of units within the population that are part of the response group, while M* signifies the number of units in the non-response group, ensuring that $M^{\prime }$  +  $M^{*}=M$. With this information, we can express the finite population CDF, *G*(*y*), in the following way:

$$G(y)=W^{\prime }G^{\prime }(y)+W^{*}G^{*}(y),$$ (1)

where, $W^{\prime }=M^{\prime }/M$, $W^{*}=M^{*}/M$ are the weights and $G^{\prime }(y)=\sum_{i=1}^{M^{\prime }}I(Y_{i}\leq y)/M^{\prime }$, $G^{*}(y)=\sum_{i=1}^{M^{*}}I(Y_{i}\leq y)/M^{*}$ correspond to CDF computed from response groups and non-response group, respectively. Here, in-dices ′ and * corresponds to response and non-response group, respectively.

*G*(*y*), of a random variable *Y* tells us the probability that *Y* could take value less than or equal to a specific value *y*. This value of *y* could be the mean, median, Quantiles or any threshold value of the random variable *Y*.

$$I(Y_{i}\leq y)=\left\{ \begin{array}{cc} 1 & \text{if}\;Y_{i}\leq y\\ 0 & \text{if}\;Y_{i}>y \end{array}\right.$$ (2)

The function $I(Y_{i}\leq y)$ is called indicator function since it indicates whether *Y*_*i*_ is less than a given value or not. The indicator function hold the same for the other variables.

Our goal is to estimate *G*(*y*), for a finite population under non-response. To achieve this, we select a sample of *m* units from a population of *M* units. Out of these *m* units, $m^{\prime }$ units respond to the survey while ${\text{m}}^{*}$ units do not respond. To obtain responses from the non-responding units, they are contacted once again by personal effort, such as telephone, email, or any other method. Then, a sub-sample of size $r=m^{*}/c$, where *c* is a number equal to or greater than 1, is obtained from the non-responding units, and it is assumed that all *r* units will respond to the survey. It should be noted that SRS without replacement is used in all stages of sampling. An unbiased estimator of *G*(*y*) under non-response can be obtained using CDF estimators, ${\hat{G}}^{\prime }(y)$ and $\hat{G}*(y)$, which are based on $m^{\prime }$ and *r*, respectively. This method is based on the work of [7] given by

$$\hat{G}(y)\;=\;w\prime {\hat{G}}^{\prime }(y)+w*\hat{G}*(y),$$ (3)

where, $w^{\prime }=m^{\prime }/m$ and w*=m*/m have their usual meanings.

It is straightforward to demonstrate that $\hat{G}(y)$ serves as an unbiased estimate of *G*(*y*). Additionally, the variability of $\hat{G}(y)$ can be stated as follows:

$$V(\hat{G}(y))\;=\;\frac{\zeta \sigma_{Y}}{m}+\frac{W*(c-1)}{m}\frac{M*\sigma_{Y}^{*}}{M*-1},$$ (4)

where, $\sigma_{Y}^{2}=G(y)(1-G(y))$, $\sigma_{Y}^{*2}=G*(y)(1-G*(y))$, and ζ=(M−m)/(M−1). For more details, see [33,9] and references cited therein.

## 3 Estimation of CDF under non-response in complex survey sampling

In this section, we will evolve unbiased estimators of *G*(*y*) for 2SCS/S2SCS and 3SCS/S3SCS under non-response. These estimators will be utilized in the subsequent sections.

### 3.1 The 2SCS with non-response

The 2SCS method selects a sample through two stages. The population comprises *N*_1_ PSUs, each with *N*_2,*i*_ SSUs. Let *Y*_*i*,*j*_ denote the characteristic value for the *j*th SSU in the *i*th PSU, where *N*_2,*i*_ is the total number of SSUs. Additionally, $N_{2,i}^{\prime }$ and $N_{2,i}^{*}$ represent the units in the response and non-response groups, such that $N_{2,i}=N_{2,i}^{\prime }+N_{2,i}^{*}$. Based on this, *G*(*y*) under 2SCS is expressed as follows:

$$G(y)=\frac{1}{N_{1}\bar{N}}\sum_{i=1}^{N_{1}}N_{2,i}G_{i}(y)=\frac{1}{N_{1}\bar{N}}\sum_{i=1}^{N_{1}}N_{2,i}(W_{i}^{\prime }G_{i}^{\prime }(y)+W_{i}^{*}G_{i}^{*}(y)),$$ (5)

where,


$$\overset{―}{N}\;=\frac{1}{N_{1}}\sum_{i=1}^{N_{1}}N_{2,i}\;,\;G_{i}^{\prime }(y)\;=\frac{1}{N_{2,i}^{\prime }}\sum_{j=1}^{N_{2,i}^{\prime }}I(Y_{i,j}\leq y)\;\text{and}\;G_{i}^{*}(y)=\frac{1}{N_{2,i}^{*}}\sum_{j=1}^{N_{2,i}^{*}}I(Y_{i,j}\leq y).$$

Here, $W_{i}^{\prime }=N_{2,i}^{\prime }/N_{2,i}$, $W_{i}^{*}=N_{2,i}^{*}/N_{2,i}$ and $G_{i}^{\prime }(y)$ and $G_{i}^{*}(y)$ correspond to the average cluster size and CDF, for the response group and non-response groups in the *i*th PSU, respectively.

To estimate finite population CDF for 2SCS while accounting for non-response, we select a sample of size *n*_1_ from *N*_1_ PSUs with SRS, and then from *i*th PSU, a sub-sample of size *n*_2,*i*_ is chosen. It is observed that out of *n*_2,*i*_ SSUs, $n_{2,i}^{\prime }$ units respond and $n_{2,i}^{*}$ units do not respond. Following [7]’s sub-sampling technique of non-respondents, we select a subsample of $r_{i}^{*}$ from the non-respondent units of $n_{2,i}^{*}$ using SRS scheme from the *i*th PSU selected in the sample such that $n_{2,i}^{*}=r_{i}^{*}/c$, where c/≥1 and then interview all $r_{i}^{*}$ units. It should be noted that the samples are chosen using SRS without replacement on all stages of sampling in the 2SCS scheme. For the 2SCS with non-response, we propose an estimator of *G*(*y*) given by:

$${\hat{G}}_{\text{2S}}^{*}(y)=\frac{1}{n\overset{―}{N}}\sum_{i=1}^{n}N_{2,i}{\hat{G}}_{i}(y)\;,$$ (6)

where,

$${\hat{G}}_{i}(y)=\;w_{i}^{\prime }{\hat{G}}_{i}^{\prime }(y)+w_{i}^{*}{\hat{G}}_{i}^{*}(y).$$ (7)

Here, $w_{i}^{\prime }=n_{2,i}^{\prime }/n_{2,i}$ and $w_{i}^{*}=n_{2,i}^{*}/n_{2,i}$ are the weights and ${\hat{G}}_{i}^{\prime }(y)$ and ${\hat{G}}_{i}^{*}(y)$ are the CDF based on $n_{2,i}^{\prime }$ and $r_{i}^{*}$ units, respectively.

In next Lemmas, we study the mathematical properties of ${\hat{G}}_{\text{2S}}^{*}(y)$.

**Lemma 1:** Under the 2SCS, ${\hat{G}}_{\text{2S}}^{*}(y)$ is an unbiased estimator of *G*(*y*).

**Proof:** By assigning indices “1”, and “2” to represent the first, and second stages of sampling, respectively, we can express it as follows:


$$E({\hat{G}}_{\text{2S}}^{*}(y))=E_{1}\left[\frac{1}{n_{1}\overset{―}{N}}\sum_{i=1}^{n_{1}}N_{2,i}E_{2}({\hat{G}}_{i}(y))\right]$$

$$=E_{1}\left[\frac{1}{n_{1}\overset{―}{N}}\sum_{i=1}^{n_{1}}N_{2,i}G_{i}(y)\right]$$

$$=\frac{1}{N_{1}\overset{―}{N}}\sum_{i=1}^{N_{1}}N_{2,i}G_{i}(y)=G(y)$$ (8)

Note that $\left(N_{2,1}G_{1}(y),N_{2,2}G_{2}(y),\ldots ,N_{2,n_{1}}G_{n_{1}}(y)\right)$ may be observed as a simple random sample of size *n*_1_ from $\left(N_{2,1}G_{1}(y),N_{2,2}G_{2}(y),\ldots ,N_{2,N_{1}}G_{N_{1}}(y)\right)$ in the first steps of sampling. For more details, see [10] and the references cited therein.

**Lemma 2:** The variances of ${\hat{G}}_{\text{2S}}^{*}(y)$ is given by


$$V({\hat{G}}_{\text{2S}}^{*}(y))=\frac{\lambda \sigma_{Y,2,b}^{2}}{n_{1}}+\frac{1}{n_{1}N_{1}}\sum_{i=1}^{N_{1}}\frac{N_{2,i}^{2}}{{\overset{―}{N}}^{2}}\left[\frac{\zeta_{i}\sigma_{Y,2,i}^{2}}{n_{2,i}}+\frac{W_{i}^{*}(c-1)}{n_{2,i}}\frac{N_{2,i}^{*}\sigma_{Y,2,i}^{*2}}{N_{2,i}^{*}-1}\right],$$

where


$$\sigma_{Y,2,b}^{2}=\frac{1}{N_{1}-1}\sum_{i=1}^{N_{1}}(N_{2,i}G_{i}(y)-\overset{―}{N}G(y){)}^{2},\;\lambda \;=\;\left(1-\frac{n_{1}}{N_{1}}\right),\;\zeta_{i}\;=\;\frac{N_{2,i}-n_{2,i}}{N_{2,i}-1},$$

$$\sigma_{Y,2,i}^{2}=G_{i}(y)(1-G_{i}(y)),\;\text{and}\;\sigma_{Y,2,i}^{*2}=G_{i}^{*}(y)(1-G_{i}^{*}(y)).$$ (9)

**Proof:** We know that

$$V({\hat{G}}_{\text{2S}}^{*}(y))=V_{1}[E_{2}({\hat{G}}_{\text{2S}}^{*}(y))]+E_{1}[V_{2}({\hat{G}}_{\text{2S}}^{*}(y))].$$ (10)

From Eq (8), $E_{2}({\hat{G}}_{\text{2S}}^{*}(y))=\sum_{i=1}^{n}N_{2,i}G_{i}(y)/(n\overset{―}{N})$. Thus by using this result:

$$V_{1}[E_{2}({\hat{G}}_{\text{2S}}^{*}(y))]=V_{1}\left(\frac{1}{n_{1}\overset{―}{N}}\sum_{i=1}^{n_{1}}N_{2,i}G_{i}(y)\right)=\frac{\lambda \sigma_{Y,2,b}^{2}}{n_{1}{\overset{―}{N}}^{2}},$$ (11)

$$E_{1}[V_{2}({\hat{G}}_{\text{2S}}^{*}(y))]=E_{1}\left[\frac{1}{n_{1}^{2}{\overset{―}{N}}^{2}}\sum_{i=1}^{n_{1}}N_{2,i}^{2}\;V_{2}({\hat{G}}_{i}(y))\right]$$

$$=\frac{1}{n_{1}N_{1}}\sum_{i=1}^{N_{1}}\frac{N_{2,i}^{2}}{{\overset{―}{N}}^{2}}\left[\frac{\zeta_{i}\sigma_{Y,2,i}^{2}}{n_{2,i}}+\frac{W_{i}^{*}(c-1)}{n_{2,i}}\frac{N_{2,i}^{*}\sigma_{Y,2,i}^{*2}}{N_{2,i}^{*}-1}\right]$$ (12)

For more details, the readers may see [33,9] and the references cited therein.

### 3.2 The S2SCS with non-response

Consider a finite population that is divided into *L* strata, with each stratum comprising *N*_1,*h*_ PSUs, where $N_{1}=\sum_{h=1}^{L}N_{1,h}$. Each PSU contains *N*_2,*i*,*h*_ SSUs for $i=1,2,\ldots ,N_{1,h}$ and h=1,2,…,L. Let *Y*_*i*,*j*,*h*_ represent the characteristic value for the *j*th SSU within the *i*th PSU of the *h*th stratum, where $j=1,2,\ldots ,N_{2,i,h}$. Furthermore, let $N_{2,i,h}^{\prime }$ and $N_{2,i,h}^{*}$ denote the counts of units in the response and non-response groups, respectively, such that $N_{2,i,h}^{\prime }\;+\;N_{2,i,h}^{*}=N_{2,i,h}$. Utilizing this information under the S2SCS framework, the CDF for finite population can be expressed as:

$$G(y)\;=\;\sum_{h=1}^{L}W_{h}G_{h}(y)\;=\;\frac{1}{\sum_{h=1}^{L}N_{1,h}{\overset{―}{N}}_{h}}\sum_{h=1}^{L}N_{1,h}{\overset{―}{N}}_{h}\;G_{h}(y),$$ (13)

where


$${\overset{―}{N}}_{h}=\frac{1}{N_{1,h}}\sum_{i=1}^{N_{1,h}}N_{2,i,h}\;\text{and}\;G_{h}(y)\;=\;\frac{1}{N_{1,h}{\overset{―}{N}}_{h}}\sum_{i=1}^{N_{1,h}}N_{2,i,h}(W_{i,h}^{\prime }G_{i,h}^{\prime }(y)+W_{i,h}^{*}G_{i,h}^{*}(y))$$

are the *h*th stratum’s average cluster sizes and CDF, respectively.

To estimate *G*(*y*) with S2SCS under non-response, *n*_1,*h*_ PSUs is chosen from the *h*th stratum. The sample sizes *n*_1,*h*_ are allotted according to an allocation system, such as equal, Neyman or proportional allocations. Further, a sub-sample of size *n*_2,*i*,*h*_ SSUs from *i*th selected PSU of *h*th stratum is selected. It should be noted that on both stages of sampling under S2SCS, the samples are taken using SRS without replacement. It is observed that out of *n*_2,*i*,*h*_ SSUs, $n_{2,i,h}^{\prime }$ units respond and $n_{2,i,h}^{*}$ units do not respond. Adopting [7] technique of sub-sampling of non-respondent, the unbiased estimator of *G*(*y*) with S2SCS can be written as:

$${\hat{G}}_{\text{S2S}}^{*}(y)=\sum_{h=1}^{L}W_{h}{\hat{G}}_{\text{2S,}h}^{*}(y),$$ (14)

where


$${\hat{G}}_{\text{2S,}h}^{*}(y)=\frac{1}{n_{1,h}{\overset{―}{N}}_{h}}\sum_{i=1}^{n_{1,h}}N_{2,i,h}{\hat{G}}_{i,h}(y)\;.$$

In the subsequent Lemmas, we will examine the mathematical properties of estimator with non-response under S2SCS.

**Lemma 3:** ${\hat{G}}_{\text{S2S}}^{*}(y)$ is an unbiased estimators of *G*(*y*). The variances of ${\hat{G}}_{\text{S2S}}^{*}(y)$ is given by

$$V({\hat{G}}_{\text{S2S}}^{*}(y))=\sum_{h=1}^{L}W_{h}^{2}\;V({\hat{G}}_{\text{2S,h}}^{*}(y)),$$ (15)

The proof is similar to Lemmas 1 and 2. The mathematical expressions of ${\hat{G}}_{\text{2S,h}}^{*}(y)$ is similar to ${\hat{G}}_{\text{2S}}^{*}(y)$ with the exception that earlier is computed from the *h*th stratum.

### 3.3 3SCS with non-response

The 3SCS extends the 2SCS method by adding a third stage. In 3SCS, the population *U* consists of *N*_1_ PSUs, each containing *N*_2,*i*_ SSUs, which in turn include *N*_3,*ij*_ TSUs. Let define *Y*_*ijk*_ as the characteristic value associated with the *k*th TSU located within the *j*th SSU of the *i*th PSU, where the indices are defined as $i=1,2,\ldots ,N_{1}$, $j=1,2,\ldots ,N_{2,i}$, and $k=1,2,\ldots ,N_{3,ij}$. Furthermore, it is established that $N_{3,ij}=N_{3,ij}^{\prime }+N_{3,ij}^{*}$, with $N_{3,ij}^{\prime }$ and $N_{3,ij}^{*}$ retaining their conventional definitions. Under 3SCS with non-response, the finite population CDF may be written as

$$G(y)\;=\;\frac{1}{N_{1}\overset{―}{T}}\sum_{i=1}^{N_{1}}\sum_{j=1}^{N_{2,i}}N_{3,ij}G_{ij}(y)\;=\;\frac{1}{N_{1}\overset{―}{T}}\sum_{i=1}^{N_{1}}\sum_{j=1}^{N_{2,i}}N_{3,ij}(W_{ij}^{\prime }G_{ij}^{\prime }(y)+W_{ij}^{*}G_{ij}^{*}(y)),$$ (16)

where


$$\overset{―}{T}\;=\;\frac{1}{N_{1}}\sum_{i=1}^{N_{1}}\sum_{j=1}^{N_{2,i}}N_{3,ij}\;\;,\;\;G_{ij}^{\prime }(y)\;=\;\frac{1}{N_{3,ij}^{\prime }}\sum_{k=1}^{N_{3,ij}^{\prime }}I(Y_{ij,k}\leq y)\;\text{and}$$

$$G_{ij}^{*}(y)\;=\;\frac{1}{N_{3,ij}^{*}}\sum_{k=1}^{N_{3,ij}^{*}}I(Y_{ij,k}\leq y).$$ (17)

In this context, $W_{ij}^{\prime }=N_{3,ij}^{\prime }/N_{3,ij}$ and $W_{ij}^{*}=N_{3,ij}^{*}/N_{3,ij}$ represent the weights, while $\overset{―}{T}$ signifies the average size of the cluster. Additionally, let $G_{ij}^{\prime }(y)$ and $G_{ij}^{*}(y)$ denote the CDF derived from the *j*th SSU of the *i*th PSU for the response group and the non-response group, respectively.

To estimate *G*(*y*) under 3SCS with non-response, an unbiased estimator of population CDF can be derived as follow. First, a sample of PSUs, *n*_1_, is chosen. Next, from each selected PSU, a sample of SSUs, *n*_2,*i*_, is drawn. Finally, within each selected SSU, a sample of TSUs, *n*_3,*ij*_, is selected. Further, let $n_{3,ij}=n_{3,ij}^{\prime }\;+\;n_{3,ij}^{*}$ where $n_{3,ij}^{\prime }$ and $n_{3,ij}^{*}$ have their usual meanings. A sub-sample of size $r_{ij}=n_{3,ij}^{*}/c$ where c≥1 units is drawn from $n_{3,ij}^{*}$ units. Under the 3SCS with non-response at third stage, an unbiased estimator of *G*(*y*) is given by:

$${\hat{G}}_{\text{3S}}^{*}(y)\;=\;\frac{1}{n_{1}\overset{―}{T}}\sum_{i=1}^{n_{1}}\frac{N_{2,i}}{n_{2,i}}\sum_{j=1}^{n_{2,i}}N_{3,ij}{\hat{G}}_{ij}(y)\;,$$ (18)

where

$${\hat{G}}_{ij}(y)\;=\;w_{ij}^{\prime }{\hat{G}}_{ij}^{\prime }(y)+w_{ij}^{*}{\hat{G}}_{ij}^{*}(y),$$ (19)

and $w_{ij}^{\prime }=n_{3,ij}^{\prime }/n_{3,ij}$, $w_{ij}^{*}=n_{3,ij}^{*}/n_{3,ij}$, have their usual meanings. ${\hat{G}}_{ij}^{\prime }(y)=\sum_{k=1}^{n_{3,ij}^{\prime }}I(Y_{ij,k}\leq y)/n_{3,ij}^{\prime }$ and ${\hat{G}}_{ij}^{*}(y)=\sum_{k=1}^{r_{ij}}I(Y_{ij,k}\leq y)/r_{ij}$ are the CDF computed from response and non-response group, respectively. It should be noted that under 3SCS with non-response, SRS without replacement is used in all stages of sampling. For more detail, see [33,1] and references cited therein.

In the subsequent Lemmas, we will examine the mathematical properties of ${\hat{G}}_{\text{3S}}^{*}(y)$.

**Lemma 4:** Under 3SCS, ${\hat{G}}_{\text{3S}}^{*}(y)$ is an unbiased estimator of *G*(*y*).

**Proof.** By assigning indices “1”, “2”, and “3” to represent the first, second, and third stages of sampling, respectively, we can express it as follows:


$$E({\hat{G}}_{\text{3S}}^{*}(y))=E_{1}E_{2}\left[\frac{1}{n_{1}\overset{―}{T}}\sum_{i=1}^{n_{1}}\frac{N_{2,i}}{n_{2,i}}\sum_{j=1}^{n_{2,i}}N_{3,ij}E_{3}({\hat{G}}_{ij}(y))\right]$$

$$=E_{1}E_{2}\left[\frac{1}{n_{1}\overset{―}{T}}\sum_{i=1}^{n_{1}}\frac{N_{2,i}}{n_{2,i}}\sum_{j=1}^{n_{2,i}}N_{3,ij}G_{ij}(y)\right]$$

$$=E_{1}\left[\frac{1}{n_{1}\overset{―}{T}}\sum_{i=1}^{n_{1}}\sum_{j=1}^{N_{2,i}}N_{3,ij}G_{ij}(y)\right]$$

$$=\frac{1}{N_{1}\overset{―}{T}}\sum_{i=1}^{N_{1}}\sum_{j=1}^{N_{2,i}}N_{3,ij}G_{ij}(y)=G(y).$$ (20)

**Lemma 5:** The variances of ${\hat{G}}_{\text{3S}}^{*}(y)$ is given by


$$V({\hat{G}}_{\text{3S}}^{*}(y))=\frac{\lambda \sigma_{Y,3,b}^{2}}{n_{1}{\overset{―}{T}}^{2}}+\frac{1}{n_{1}N_{1}{\overset{―}{T}}^{2}}\sum_{i=1}^{N_{1}}\frac{\lambda_{i}N_{2,i}^{2}\sigma_{Y,3,i}^{2}}{n_{2,i}}+\frac{1}{n_{1}N_{1}}\sum_{i=1}^{N_{1}}\frac{N_{2,i}}{n_{2,i}}\sum_{j=1}^{N_{2,i}}$$

$$\frac{N_{3,ij}^{2}}{{\overset{―}{T}}^{2}}\left[\frac{\lambda_{ij}\sigma_{Y,3,ij}^{2}}{n_{3,ij}}+\frac{W_{ij}^{*}(c-1)}{n_{3,ij}}\frac{N_{3,ij}^{*}\sigma_{Y,3,ij}^{*2}}{N_{3,ij}^{*}-1}\right]$$ (21)

where


$$\sigma_{Y,3,b}^{2}=\frac{1}{N_{1}-1}\sum_{i=1}^{N_{1}}(N_{2,i}G_{i}(y)-\overset{―}{N}G(y){)}^{2},\;\sigma_{Y,i}^{2}\;=\;\frac{1}{N_{2,i}-1}\sum_{j=1}^{N_{2,i}}{\left(N_{3,ij}G_{ij}(y)-G_{i}(y)\right)}^{2},$$

$$G_{i}(y)=\frac{1}{N_{2,i}}\sum_{j=1}^{N_{2,i}}N_{3,ij}G_{ij}(y),\;\lambda_{i}\;=\;\left(1-\frac{n_{2,i}}{N_{2,i}}\right)\;\;\lambda_{ij}\;=\;\frac{N_{3,ij}-n_{3,ij}}{N_{3,ij}-1},$$

$$\sigma_{Y,3,ij}^{2}=G_{ij}(y)(1-G_{ij}(y))\;\text{and}\;\sigma_{Y,3,ij}^{*2}=G_{ij}^{*}(y)(1-G_{ij}^{*}(y)).$$ (22)

**Proof:**

$$V({\hat{G}}_{\text{3S}}^{*}(y))=E_{1}E_{2}V_{3}[{\hat{G}}_{\text{3S}}^{*}(y)]+E_{1}V_{2}E_{3}[{\hat{G}}_{\text{3S}}^{*}(y)]+V_{1}E_{2}E_{3}[{\hat{G}}_{\text{3S}}^{*}(y)].$$ (23)

From Eq (20), we can write


$$V_{1}E_{2}E_{3}[{\hat{G}}_{\text{3S}}^{*}(y)]=V_{1}E_{2}\left[\frac{1}{n_{1}\overset{―}{T}}\sum_{i=1}^{n_{1}}\frac{N_{2,i}}{n_{2,i}}\sum_{j=1}^{n_{2,i}}N_{3,ij}G_{ij}(y)\right]$$

$$=V_{1}\left(\frac{1}{n_{1}\overset{―}{T}}\sum_{i=1}^{n_{1}}N_{2,i}G_{i}(y)\right)=\frac{\lambda \sigma_{Y,3,b}^{2}}{n_{1}{\overset{―}{T}}^{2}}$$ (24)

and


$$E_{1}V_{2}E_{3}[{\hat{G}}_{\text{3S}}^{*}(y)]=E_{1}V_{2}\left[\frac{1}{n_{1}\overset{―}{T}}\sum_{i=1}^{n_{1}}\frac{N_{2,i}}{n_{2,i}}\sum_{j=1}^{n_{2,i}}N_{3,ij}G_{ij}(y)\right]$$

$$=E_{1}\left[\frac{1}{n_{1}^{2}{\overset{―}{T}}^{2}}\sum_{i=1}^{n_{1}}N_{2,i}^{2}\;V_{2}(\frac{1}{n_{2,i}}\sum_{j=1}^{n_{2,i}}N_{3,ij}G_{ij}(y))\right]$$

$$=\frac{1}{n_{1}N_{1}{\overset{―}{T}}^{2}}\sum_{i=1}^{N_{1}}\frac{\lambda_{i}N_{2,i}^{2}\sigma_{Y,3,i}^{2}}{n_{2,i}}.$$ (25)

Also from Eq (20), we have


$$V_{3}[{\hat{G}}_{\text{3S}}^{*}(y)]=\frac{1}{n_{1}^{2}{\overset{―}{T}}^{2}}\sum_{i=1}^{n_{1}}\frac{N_{2,i}^{2}}{n_{2,i}^{2}}\sum_{j=1}^{n_{2,i}}N_{3,ij}^{2}\;V_{3}({\hat{G}}_{ij}(y))$$

$$=\frac{1}{n_{1}^{2}}\sum_{i=1}^{n_{1}}\frac{N_{2,i}^{2}}{n_{2,i}^{2}}\sum_{j=1}^{n_{2,i}}\frac{N_{3,ij}^{2}}{{\overset{―}{T}}^{2}}\;\left[\frac{\lambda_{ij}\sigma_{Y,3,ij}^{2}}{n_{3,ij}}+\frac{W_{ij}^{*}(c-1)}{n_{3,ij}}\frac{N_{3,ij}^{*}\sigma_{Y,3,ij}^{*2}}{N_{3,ij}^{*}-1}\right].$$ (26)

Now take expectations of Eq (20) to get

$$E_{1}E_{2}V_{3}[{\hat{G}}_{\text{3S}}^{*}(y)]\;=\;\frac{1}{n_{1}N_{1}{\overset{―}{T}}^{2}}\sum_{i=1}^{N_{1}}\frac{N_{2,i}}{n_{2,i}}\sum_{j=1}^{N_{2,i}}\frac{N_{3,ij}^{2}}{{\overset{―}{T}}^{2}}\;\left[\frac{\lambda_{ij}\sigma_{Y,3,ij}^{2}}{n_{3,ij}}+\frac{W_{ij}^{*}(c-1)}{n_{3,ij}}\frac{N_{3,ij}^{*}\sigma_{Y,3,ij}^{*2}}{N_{3,ij}^{*}-1}\right]$$ (27)

Now Eq (21) follows by replacing Eq (24), Eq (25) and Eq (27) in Eq (23), which completes the proof.

### 3.4 S3SCS under Non-Response

S3SCS begins by dividing the entire population *U* into *L* strata, based on certain characteristics such as different regions or income levels. Within the *h*th stratum, the population contains *N*_1,*h*_ PSUs, each with *N*_2,*i*,*h*_ SSUs, each of which has *N*_3,*ij*,*h*_ TSUs for h=1,2,⋯,L. Let *Y*_*ijk*,*h*_ be the characteristics under study obtained for the *k*th TSUs in the *j*th SSUs drawn from the *i*th PSUs within the *h*th stratum for $i=1,2,\ldots ,N_{1,h}$, $j=1,2,\ldots ,N_{2,i,h}$, and $k=1,2,\ldots ,N_{3,ij,h}$. Further, let $N_{3,ij,h}=N_{3,ij,h}^{\prime }\;+\;N_{3,ij,h}^{*}$ where $N_{3,ij,h}^{\prime }$ and $N_{3,ij,h}^{*}$ have their usual meanings. Under S3SCS with non-response the population CDF may be written as:


$$G(y)=\sum_{h=1}^{L}\frac{W_{h}}{N_{1,h}{\overset{―}{T}}_{h}}\sum_{i=1}^{N_{1,h}}\sum_{j=1}^{N_{2,i,h}}N_{3,ij,h}(W_{ij,h}^{\prime }G_{ij,h}^{\prime }(y)+W_{ij,h}^{*}G_{ij,h}^{*}(y)),$$

$$=\sum_{h=1}^{L}\frac{W_{h}}{N_{1,h}{\overset{―}{T}}_{h}}\sum_{i=1}^{N_{1,h}}\sum_{j=1}^{N_{2,i,h}}N_{3,ij,h}G_{ij,h}(y),$$

$$=\sum_{h=1}^{L}W_{h}G_{h}(y),$$

$$=\frac{1}{\sum_{h=1}^{L}N_{1,h}{\overset{―}{T}}_{h}}\sum_{h=1}^{L}N_{1,h}{\overset{―}{T}}_{h}G_{h}(y).$$ (28)

In estimating the population CDF using S3SCS under non-response conditions, the sampling process consists of several stages within each stratum. First, a sample of PSUs is selected from *N*_1,*h*_, resulting in a size of *n*_1,*h*_. From each selected PSU, SSUs are sampled from *N*_2,*i*,*h*_, yielding a size of *n*_2,*i*,*h*_. Lastly, TSUs are chosen from each sampled SSU, with a size of *n*_3,*ij*,*h*_ from *N*_3,*ij*,*h*_. It is observed that out of *n*_3,*ij*,*h*_, $n_{3,ij,h}^{\prime }$ units respond and $n_{3,ij,h}^{*}$ units do not respond, such that $n_{3,ij,h}=n_{3,ij,h}^{\prime }+n_{3,ij,h}^{*}$. Adopting [8] technique of sub-sampling of non-respondent, the unbiased estimator of population CDF, *G*(*y*), under S3SCS with non-response can be written as:

$${\hat{G}}_{\text{S3S}}^{*}(y)\;=\;\sum_{h=1}^{L}W_{h}{\hat{G}}_{\text{3S,}h}^{*}(y),$$ (29)

where


$${\hat{G}}_{\text{3S,}h}^{*}(y)=\frac{1}{n_{1,h}\overset{―}{T_{h}}}\sum_{i=1}^{n_{1,h}}\frac{N_{2,i,h}}{n_{2,i,h}}\sum_{j=1}^{n_{2,i,h}}N_{3,ij,h}{\hat{G}}_{ij,h}(y),\;\;\text{where}$$

$${\hat{G}}_{ij,h}(y)=w_{ij,h}^{\prime }{\hat{G}}_{ij,h}^{\prime }(y)+w_{ij,h}^{*}{\hat{G}}_{ij,h}^{*}(y).$$

Here, $w_{ij,h}^{\prime }=n_{3,ij,h}^{\prime }/n_{3,ij,h}$ and $w_{ij,h}^{*}=n_{3,ij,h}^{*}/n_{3,ij,h}$ have there usual meanings.

In the forthcoming Lemmas, we examine the characteristics of the CDF estimator in the context of non-response within the S3SCS framework.

**Lemma 6:** ${\hat{G}}_{\text{S3S}}^{*}(y)$ is an unbiased estimators of *G*(*y*). The variances of ${\hat{G}}_{\text{S3S}}^{*}(y)$ is given by

$$V({\hat{G}}_{\text{S3S}}^{*}(y))=\sum_{h=1}^{L}W_{h}^{2}\;V({\hat{G}}_{\text{3S,}h}^{*}(y))$$ (30)

**Proof.** The proof has a resemblance to Lemmas 4 and 5. The proof of $V({\hat{G}}_{\text{3S,}h}^{*}(y))$ is completed by observing that its mathematical expressions are similar to $V({\hat{G}}_{\text{3S}}^{*}(y))$, except that the earlier is assessed from the *h*th stratum.

## 4 Covariance computation and estimation in the presence of non-response

Here, we use the previously discussed complex survey sampling schemes to formulate precise mathematical equations for the covariance of CDF estimators on the basis of non-response given above in Section 3.

### 4.1 Two-stage and stratified two-stage cluster sampling

In a finite population *U*, we assume that *X* represents the auxiliary, and *Y* denotes the primary variable under study. Additionally, to approximate (*G*(*y*),*G*(*x*)) within the frameworks of 2SCS and S2SCS, let $\left({\hat{G}}_{\text{2S}}^{*}(y),{\hat{G}}_{\text{2S}}^{*}(x)\right)$ and $\left({\hat{G}}_{\text{S2S}}^{*}(y),{\hat{G}}_{\text{S2S}}^{*}(x)\right)$ represent the corresponding CDF estimators derived from variables (*Y*,*X*). It should be noted that the CDF estimators on the basis of *X* can be computed on the similar lines of *Y*.

**Lemma 7:** Under 2SCS scheme, the covariance between ${\hat{G}}_{\text{2S}}^{*}(y)$ and ${\hat{G}}_{\text{2S}}^{*}(x)$ is given by

$$C({\hat{G}}_{\text{2S}}^{*}(y),{\hat{G}}_{\text{2S}}^{*}(x))=\frac{\lambda \sigma_{XY,2b}}{n_{1}{\overset{―}{N}}^{2}}+\frac{1}{n_{1}N_{1}}\sum_{i=1}^{N_{1}}\frac{N_{2,i}^{2}}{{\overset{―}{N}}^{2}}\left[\frac{\lambda_{i}\sigma_{XY,2i}}{n_{2,i}}+\frac{W_{i}^{*}(c-1)\sigma_{XY,2i}^{*}}{n_{2,i}}\right],$$ (31)

where

$$\sigma_{XY,2,b}=\frac{1}{N_{1}-1}\sum_{i=1}^{N_{1}}((N_{2,i}G_{i}(y)-\overset{―}{N}G(y))(N_{2,i}G_{i}(x)-\overset{―}{N}G(x))),$$ (32)

$$\sigma_{XY,2,i}=\frac{1}{N_{2,i}-1}\sum_{j=1}^{N_{2,i}}((I(Y_{i,j}\leq y)-G_{i}(y))(I(X_{i,j}\leq x)-G_{i}(x))),$$ (33)

$$\sigma_{XY,2,i}^{*}=\frac{1}{N_{2,i}^{*}-1}\sum_{j=1}^{N_{2,i}^{*}}((I(Y_{i,j}\leq y)-G_{i}^{*}(y))(I(X_{i,j}\leq x)-G_{i}^{*}(x))),$$ (34)

**Proof.** Here, $\sigma_{XY,2b}$, $\sigma_{XY,2i}$, and $\sigma_{XY,2i}^{*}$ retain their standard interpretations. The demonstration of this Lemma can be found in the S3 Appendix.

**Lemma 8:** Under S2SCS scheme, the covariance between ${\hat{G}}_{\text{S2S}}^{*}(y)$ and ${\hat{G}}_{\text{S2S}}^{*}(x)$ is given by

$$C({\hat{G}}_{\text{S2S}}^{*}(y),{\hat{G}}_{\text{S2S}}^{*}(x))=\sum_{h=1}^{L}W_{h}^{2}\;C({\hat{G}}_{\text{2S,}h}^{*}(y),{\hat{G}}_{\text{2S,}h}^{*}(x)),$$ (35)

**Proof.** The proof has a resemblance to Lemmas 7. The proof of $C({\hat{G}}_{\text{S2S}}^{*}(y),{\hat{G}}_{\text{S2S}}^{*}(x))$ is completed by observing that its mathematical expressions $C({\hat{G}}_{\text{2S,}h}^{*}(y),{\hat{G}}_{\text{2S,}h}^{*}(x))$ is similar to $C({\hat{G}}_{\text{2S}}^{*}(y),{\hat{G}}_{\text{2S}}^{*}(x))$, except that the aforesaid is assessed from the *h*th stratum.

### 4.2 Three-stage and stratified three-stage cluster sampling

Under 3SCS and S3SCS with non response, let $\left({\hat{G}}_{\text{3S}}^{*}(y),{\hat{G}}_{\text{3S}}^{*}(x)\right)$ and $\left({\hat{G}}_{\text{S3S}}^{*}(y),{\hat{G}}_{\text{S3S}}^{*}(x)\right)$ be the respective CDF estimators of (*G*(*y*),*G*(*x*)) that are based on (*Y*,*X*), respectively.

**Lemma 9:** Under 3SCS scheme, the covariance between ${\hat{G}}_{\text{3S}}^{*}(y)$ and ${\hat{G}}_{\text{3S}}^{*}(x)$ is given by

$$C({\hat{G}}_{\text{3S}}^{*}(y),{\hat{G}}_{\text{3S}}^{*}(x))=\frac{\lambda \sigma_{XY,3,b}^{2}}{n_{1}{\overset{―}{T}}^{2}}+\frac{1}{n_{1}N_{1}{\overset{―}{T}}^{2}}\sum_{i=1}^{N_{1}}\frac{\lambda_{i}N_{2,i}^{2}\sigma_{XY,3,i}^{2}}{n_{2,i}}+\frac{1}{n_{1}N_{1}}\sum_{i=1}^{N}\frac{N_{2,i}}{n_{2,i}}\sum_{j=1}^{N_{2,i}}\;\frac{N_{3,ij}^{2}}{{\overset{―}{T}}^{2}}\left[\frac{\lambda_{ij}\sigma_{XY,3,ij}}{n_{3,ij}}+\frac{W_{ij}^{*}(c-1)\sigma_{XY,3,ij}^{*}}{n_{3,ij}}\right]$$ (36)

where

$$\sigma_{XY,3,b}=\frac{1}{N_{1}-1}\sum_{i=1}^{N_{1}}((N_{2,i}G_{i}(y)-\overset{―}{T}G(y))(N_{2,i}G_{i}(x)-\overset{―}{T}G(x))),$$ (37)

$$\sigma_{XY,3,i}=\frac{1}{N_{2,i}-1}\sum_{j=1}^{N_{2,i}}((N_{3,ij}G_{ij}(y)-G_{i}(y))(N_{3,ij}G_{ij}(x)-G_{i}(x))),$$ (38)

$$\sigma_{XY,3,ij}=\frac{1}{N_{3,ij}-1}\sum_{k=1}^{N_{3,ij}}((I(Y_{ij,k}\leq y)-G_{ij}(y))(I(X_{ij,k}\leq x)-G_{ij}(x))),$$ (39)

$$\sigma_{XY,3,ij}^{*}=\frac{1}{N_{3,ij}^{*}-1}\sum_{k=1}^{N_{3,ij}^{*}}((I(Y_{ij,k}\leq y)-G_{ij}^{*}(y))(I(X_{ij,k}\leq x)-G_{ij}^{*}(x))),$$ (40)

and $\lambda_{ij}=1-n_{3,ij}/N_{3,ij}$.

**Proof.** Here, $\sigma_{XY,3,b}$, $\sigma_{XY,3,i}$, $\sigma_{XY,3,ij}$ and $\sigma_{XY,3,ij}^{*}$ retain their standard interpretations. The demonstration of this Lemma can be accessed in the S3 Appendix.

**Lemma 10:** Under S3SCS scheme, the covariance between ${\hat{G}}_{\text{S3S}}^{*}(y)$ and ${\hat{G}}_{\text{S3S}}^{*}(x)$ is given by

$$C({\hat{G}}_{\text{S3S}}^{*}(y),{\hat{G}}_{\text{S3S}}^{*}(x))=\sum_{h=1}^{L}W_{h}^{2}\;C({\hat{G}}_{\text{3S,}h}^{*}(y),{\hat{G}}_{\text{3S,}h}^{*}(x))$$ (41)

**Proof.** The proof has a resemblance to Lemmas 9. The proof of $C({\hat{G}}_{\text{S3S}}^{*}(y),{\hat{G}}_{\text{S3S}}^{*}(x))$ is completed by observing that its mathematical expressions $C({\hat{G}}_{\text{3S,}h}^{*}(y),{\hat{G}}_{\text{3S,}h}^{*}(x))$ is similar to $C({\hat{G}}_{\text{3S}}^{*}(y),{\hat{G}}_{\text{3S}}^{*}(x))$, except that the earlier is computed from the *h*th stratum.

## 5 CDF estimation under non-response using auxiliary information

In this section, two families of estimators, namely ratio/product and exponential ratio/product, are developed, which utilize auxiliary information to approximate the CDF of the population, *G*(*y*), in the context of complex survey sampling scheme with non-response. To acquire the biases and MSEs of the suggested families of estimators for *G*(*y*), we could look into the following relative error terms: Let $\varepsilon_{0}$ represents the relative error associated with the estimation of *G*(*y*), which is defined as:


$$\varepsilon_{0}\;=\;\frac{{\hat{G}}_{\text{S}}^{*}(y)-G(y)}{G(y)}\;\text{and}\;$$

let $\varepsilon_{1}$ represents the relative error associated with the estimation of *G*(*x*), which is defined as:

$$\varepsilon_{1}\;=\;\frac{{\hat{G}}_{\text{S}}^{*}(x)-G(x)}{G(x)},$$ (42)

such that $E(\varepsilon_{0})=E(\varepsilon_{1})=0$. Furthermore, we define the variance and covariance parameters as follows:

$$\Lambda_{rs}=E\left(\varepsilon_{0}^{r}\varepsilon_{1}^{s}\right)\;=\;E\left[{\left(\frac{{\hat{G}}_{\text{S}}^{*}(y)-G(y)}{G(y)}\right)}^{r}{\left(\frac{{\hat{G}}_{\text{S}}^{*}(x)-G(x)}{G(x)}\right)}^{s}\right],$$ (43)

which gives


$$\Lambda_{20}\;=\;E(\varepsilon_{0}{)}^{2}\;=\;E{\left(\frac{{\hat{G}}_{\text{S}}^{*}(y)-G(y)}{G(y)}\right)}^{2}\;=\;\frac{V({\hat{G}}_{\text{S}}^{*}(y))}{(G(y){)}^{2}},$$

$$\Lambda_{02}\;=\;E(\varepsilon_{1}{)}^{2}\;=\;E{\left(\frac{{\hat{G}}_{\text{S}}^{*}(x)-G(x)}{G(x)}\right)}^{2}\;=\;\frac{V({\hat{G}}_{\text{S}}^{*}(x))}{(G(x){)}^{2}},$$

$$\Lambda_{11}\;=\;E(\varepsilon_{0}\varepsilon_{1})=E\left[\left(\frac{{\hat{G}}_{\text{S}}^{*}(y)-G(y)}{G(y)}\right)\left(\frac{{\hat{G}}_{\text{S}}^{*}(x)-G(x)}{G(x)}\right)\right]\;=\;\frac{C({\hat{G}}_{\text{S}}^{*}(y),{\hat{G}}_{\text{S}}^{*}(x))}{G(y)\;G(x)},$$

where, $\Lambda_{20}$ represents the relative variance of the estimator for *G*(*y*), quantifying the dispersion of the estimated CDF around its true value, normalized by $(\text{G}(\text{y}){)}^{2}$. $\Lambda_{02}$ represents the relative variance of the estimator for *G*(*x*), similarly normalized by $(\text{G}(\text{x}){)}^{2}$. $\Lambda_{11}$ represents the relative variance of the estimator of *G*(*y*) and *G*(*x*), normalized by *G*(*y*)*G*(*x*), indicating the degree of association between the two estimators. ${\hat{G}}_{\text{S}}^{*}(y)$ represent a CDF estimator derived from the sampling scheme S, where S corresponds to 2S, 3S, S2S, or S3S.

### 5.1 First proposed family of CDF estimators

We suggest a class of ratio and product-type estimators, analogous to those introduced by [1] and [14], for estimating the population CDF under non-response.

$${\hat{G}}_{R}^{*}(y)\;=\;{\hat{G}}_{\text{S}}^{*}(y){\left(\frac{aG(x)+b}{\eta (a{\hat{G}}_{\text{S}}^{*}(x)+b)+(1-\eta )(aG(x)+b)}\right)}^{\psi },$$ (44)

where *a* is different from 0 and *b* as real numbers or functions of the known parameters of the supplementary variable *X* such as coefficient of variation $\left(CV_{X}\right)$, correlation coefficient $\left(\rho_{XY}\right)$, coefficient of skewness $\left(\beta_{1,X}\right)$ and coefficient of kurtosis $\left(\beta_{2,X}\right)$ etc. The selected scalars, ψ(=1,−1) and η(0≤η≤1), minimize the MSE of ${\hat{G}}_{R}^{*}(y)$. Different estimators of ${\hat{G}}_{R}^{*}(y)$ may be developed by selecting appropriate *a*, *b*, ψ, and η values. In Table 1, we display some members of ${\hat{G}}_{R}^{*}(y)$ for various values of *a*, *b*, η, and ψ.

**Table 1:** Several members of the suggested families of CDF estimators under non-response

${\hat{G}}_{R}^{*}(y)$	${\hat{G}}_{E}^{*}(y)$	ψ	η	*a*	*b*
${\hat{G}}_{R}^{*(1)}(y)={\hat{G}}_{\text{S}}^{*}(y)\left(\frac{G(x)}{{\hat{G}}_{\text{S}}^{*}(x)}\right)$	${\hat{G}}_{E}^{*(1)}(y)={\hat{G}}_{\text{S}}^{*}(y)\;\text{exp}\left(\frac{G(x)-{\hat{G}}_{\text{S}}^{*}(x)}{G(x)+{\hat{G}}_{\text{S}}^{*}(x)}\right)$	1	1	1	0
${\hat{G}}_{R}^{*(2)}(y)={\hat{G}}_{\text{S}}^{*}(y)\left(\frac{G(x)+\rho_{XY}}{{\hat{G}}_{\text{S}}^{*}(x)+\rho_{XY}}\right)$	${\hat{G}}_{E}^{*(2)}(y)={\hat{G}}_{\text{S}}^{*}(y)\;\text{exp}\left(\frac{G(x)-{\hat{G}}_{\text{S}}^{*}(x)}{G(x)+{\hat{G}}_{\text{S}}^{*}(x)+2\rho_{XY}}\right)$	1	1	1	$\rho_{XY}$
${\hat{G}}_{R}^{*(3)}(y)={\hat{G}}_{\text{S}}^{*}(y)\left(\frac{G(x)+CV_{X}}{{\hat{G}}_{\text{S}}^{*}(x)+CV_{X}}\right)$	${\hat{G}}_{E}^{*(3)}(y)={\hat{G}}_{\text{S}}^{*}(y)\;\text{exp}\left(\frac{G(x)-{\hat{G}}_{\text{S}}^{*}(x)}{G(x)+{\hat{G}}_{\text{S}}^{*}(x)+2CV_{X}}\right)$	1	1	1	$CV_{X}$
${\hat{G}}_{R}^{*(4)}(y)={\hat{G}}_{\text{S}}^{*}(y)\left(\frac{G(x)+\beta_{2,X}}{{\hat{G}}_{\text{S}}^{*}(x)+\beta_{2,X}}\right)$	${\hat{G}}_{E}^{*(4)}(y)={\hat{G}}_{\text{S}}^{*}(y)\;\text{exp}\left(\frac{G(x)-{\hat{G}}_{\text{S}}^{*}(x)}{G(x)+{\hat{G}}_{\text{S}}^{*}(x)+2\beta_{2,X}}\right)$	1	1	1	$\beta_{2,X}$
${\hat{G}}_{R}^{*(5)}(y)={\hat{G}}_{\text{S}}^{*}(y)\left(\frac{CV_{X}G(x)+\beta_{2,X}}{CV_{X}{\hat{G}}_{\text{S}}^{*}(x)+\beta_{2,X}}\right)$	${\hat{G}}_{E}^{*(5)}(y)={\hat{G}}_{\text{S}}^{*}(y)\;\text{exp}\left(\frac{CV_{X}(G(x)-{\hat{G}}_{\text{S}}^{*}(x))}{CV_{X}(G(x)+{\hat{G}}_{\text{S}}^{*}(x))+2\beta_{2,X}}\right)$	1	1	$CV_{X}$	$\beta_{2,X}$
${\hat{G}}_{R}^{*(6)}(y)={\hat{G}}_{\text{S}}^{*}(y)\left(\frac{\beta_{2,X}G(x)+CV_{X}}{\beta_{2,X}{\hat{G}}_{\text{S}}^{*}(x)+CV_{X}}\right)$	${\hat{G}}_{E}^{*(6)}(y)={\hat{G}}_{\text{S}}^{*}(y)\;\text{exp}\left(\frac{\beta_{2,X}(G(x)-{\hat{G}}_{\text{S}}^{*}(x))}{\beta_{2,X}(G(x)+{\hat{G}}_{\text{S}}^{*}(x))+2CV_{X}}\right)$	1	1	$\beta_{2,X}$	$CV_{X}$
${\hat{G}}_{R}^{*(7)}(y)={\hat{G}}_{\text{S}}^{*}(y)\left(\frac{\rho_{XY}G(x)+CV_{X}}{\rho_{XY}{\hat{G}}_{\text{S}}^{*}(x)+CV_{X}}\right)$	${\hat{G}}_{E}^{*(7)}(y)={\hat{G}}_{\text{S}}^{*}(y)\;\text{exp}\left(\frac{\rho_{XY}(G(x)-{\hat{G}}_{\text{S}}^{*}(x))}{\rho_{XY}(G(x)+{\hat{G}}_{\text{S}}^{*}(x))+2CV_{X}}\right)$	1	1	$\rho_{XY}$	$CV_{X}$
${\hat{G}}_{R}^{*(8)}(y)={\hat{G}}_{\text{S}}^{*}(y)\left(\frac{CV_{X}G(x)+\rho_{XY}}{CV_{X}{\hat{G}}_{\text{S}}^{*}(x)+\rho_{XY}}\right)$	${\hat{G}}_{E}^{*(8)}(y)={\hat{G}}_{\text{S}}^{*}(y)\;\text{exp}\left(\frac{CV_{X}(G(x)-{\hat{G}}_{\text{S}}^{*}(x))}{CV_{X}(G(x)+{\hat{G}}_{\text{S}}^{*}(x))+2\rho_{XY}}\right)$	1	1	$CV_{X}$	$\rho_{XY}$
${\hat{G}}_{R}^{*(9)}(y)={\hat{G}}_{\text{S}}^{*}(y)\left(\frac{G(x)+\beta_{1,X}}{{\hat{G}}_{\text{S}}^{*}(x)+\beta_{1,X}}\right)$	${\hat{G}}_{E}^{*(9)}(y)={\hat{G}}_{\text{S}}^{*}(y)\;\text{exp}\left(\frac{G(x)-{\hat{G}}_{\text{S}}^{*}(x)}{G(x)+{\hat{G}}_{\text{S}}^{*}(x)+2\beta_{1,X}}\right)$	1	1	1	$\beta_{1,X}$

The bias and MSE of ${\hat{G}}_{R}^{*}(y)$ can be approximated mathematically by writing ${\hat{G}}_{\text{S}}^{*}(y)=G(y)(1+\varepsilon_{0})$ and ${\hat{G}}_{\text{S}}^{*}(x)=G(x)(1+\varepsilon_{1})$. The expression on right-hand side (RHS) of Eq (47) can be written in terms of ε’s:

$${\hat{G}}_{R}^{*}(y)=G(y)(1+\varepsilon_{0})(1+\eta \upsilon \varepsilon_{1}{)}^{-\psi },$$ (45)

where υ=aG(x)/(aG(x)  +  b). By expanding the RHS of Eq (48) and considering only the terms of order second power in ε’s, we have

$${\hat{G}}_{R}^{*}(y)\;\approx \;G(y)\left(1+\varepsilon_{0}-\eta \upsilon \psi \varepsilon_{1}+\frac{\psi (\psi +1)}{2}\eta^{2}\upsilon^{2}\varepsilon_{1}^{2}-\eta \upsilon \psi \varepsilon_{0}\varepsilon_{1}\right)$$ (46)

To obtain bias of ${\hat{G}}_{R}^{*}(y)$, subtract *G*(*y*) from both sides of Eq (49) and consider only the terms up-to first order of approximation, given as:

$$\text{Bias}({\hat{G}}_{R}^{*}(y))\;\approx \;G(y)\left(\frac{\psi (\psi +1)}{2}\;\eta^{2}\upsilon^{2}\Lambda_{02}-\eta \upsilon \psi \Lambda_{11}\right).$$ (47)

Retain terms up-to first order of approximation, we can write Eq (49) as follows:

$${\hat{G}}_{R}^{*}(y)-G(y)\approx G(y)(\varepsilon_{0}-\eta \upsilon \psi \varepsilon_{1})$$ (48)

To obtain MSE of ${\hat{G}}_{R}^{*}(y)$, take square and then its expectation on both sides of the Eq (51) and retain terms up-to first order, we have:

$$\text{MSE}({\hat{G}}_{R}^{*}(y))\;\approx \;G(y{)}^{2}\left(\Lambda_{20}+\eta^{2}\upsilon^{2}\psi^{2}\Lambda_{02}-2\eta \upsilon \psi \Lambda_{11}\right),$$ (49)

The minimal MSE of ${\hat{G}}_{R}^{*}(y)$ is given by

$${\text{MSE}}_{\text{min}}({\hat{G}}_{R}^{*}(y))\approx G(y{)}^{2}\left(\Lambda_{20}-\frac{V_{11}^{2}}{\Lambda_{02}}\right)$$ (50)

$$\approx G(y{)}^{2}\Lambda_{20}\;(1-\kappa^{2}),$$ (51)

where, $\kappa =\Lambda_{11}/\sqrt{\Lambda_{20}\Lambda_{02}}$ is the correlation coefficient between ${\hat{G}}_{\text{S}}^{*}(y)$ and ${\hat{G}}_{\text{S}}^{*}(x)$. The minimum MSE of ${\hat{G}}_{R}^{*}(y)$ can be obtained by utilizing the optimum value of (ηυψ), say $(\eta \upsilon \psi {)}_{\text{opt}}=\Lambda_{11}/\Lambda_{02}$.

### 5.2 Second proposed family of CDF estimators

We propose another class of exponential ratio/product estimators similar to those proposed by [1] and [28] to estimate the population CDF *G*(*y*) under non-response given as:

$${\hat{G}}_{E}^{*}(y)\;=\;{\hat{G}}_{\text{S}}^{*}(y)\;\text{exp}\left(\frac{\left(a\psi G(x)+b)-(a\psi {\hat{G}}_{\text{S}}^{*}(x)+b\right)}{\left(aG(x)+b)+(a{\hat{G}}_{\text{S}}^{*}(x)+b\right)}\right),$$ (52)

where *a*, *b*, and ψ have their usual meanings. Some members of ${\hat{G}}_{E}^{*}(y)$ for various values of *a*, *b*, η, and ψ can be seen in Table 1. The bias and MSE of ${\hat{G}}_{E}^{*}(y)$ can be obtained by expressing the Eq (55) in terms of ε’s as:


$${\hat{G}}_{E}^{*}(y)=G(y)(1+\varepsilon_{0})\;\text{exp}\left(\frac{a\psi G(x)-a\psi G(x)(1+\varepsilon_{1})}{aG(x)+2b+aG(x)(1+\varepsilon_{1})}\right)$$

$$=G(y)(1+\varepsilon_{0})\;\text{exp}\left(-\tau \psi \varepsilon_{1}(1+\tau \varepsilon_{1}{)}^{-1}\right),$$ (53)

where τ=aG(x)/(2aG(x)+2b). By expanding the RHS of Eq (56) and considering only the terms of order second power in ε’s, we have

$${\hat{G}}_{E}^{*}(y)\;\approx \;G(y)\left(1+\varepsilon_{0}-\tau \psi \varepsilon_{1}+\frac{\psi (\psi +1)}{2}\;\tau^{2}\varepsilon_{1}^{2}-\tau \psi \varepsilon_{0}\varepsilon_{1}\right).$$ (54)

To obtain bias of ${\hat{G}}_{E}^{*}(y)$, subtract *G*(*y*) from both sides of Eq (57) and consider only the terms up-to first order of approximation, given as:

$$\text{Bias}({\hat{G}}_{E}^{*}(y))\;\approx \;G(y)\left(\frac{\psi (\psi +1)}{2}\;\tau^{2}\Lambda_{02}-\tau \psi \Lambda_{11}\right).$$ (55)

Retain terms up-to first order of approximation, we can write Eq (57) as follows:

$${\hat{G}}_{E}^{*}(y)-G(y)\approx G(y)(\varepsilon_{0}-\tau \psi \varepsilon_{1}).$$ (56)

Under the first order of approximation, we obtain the MSE of ${\hat{G}}_{E}^{*}(y)$ by taking the square on both sides of Eq (59).

$$\text{MSE}({\hat{G}}_{E}^{*}(y))\approx G(y{)}^{2}\left(\Lambda_{20}+\tau^{2}\psi^{2}\Lambda_{02}-2\tau \psi \Lambda_{11}\right).$$ (57)

The minimal MSE of ${\hat{G}}_{E}^{*}(y)$ can be expressed by utilizing the optimum value of (τψ), say $(\tau \psi {)}_{\text{opt}}=\Lambda_{11}/\Lambda_{02}$, is given by

$${\text{MSE}}_{\text{min}}({\hat{G}}_{E}^{*}(y))\approx G(y{)}^{2}\Lambda_{20}\;(1-\kappa^{2}),$$ (58)

which is equivalent to that of ${\text{MSE}}_{\text{min}}({\hat{G}}_{R}^{*}(y))$.

A variety of estimators can be derived from the suggested families ${\hat{G}}_{R}(y)$ and ${\hat{G}}_{E}(y)$, as shown in Eqs (47) and (55), by selecting appropriate values for the parameters *a*, *b*, η, and ψ. By imputing specific values of η, ψ, *a*, and *b* into Eqs (50), (52), (58), and (60), we can derive the first-order approximations of the bias and MSE/variance for the respective members of the families ${\hat{F}}_{R}(y)$ and ${\hat{F}}_{E}(y)$.

### 5.3 Difference CDF Estimator

Additionally, supplementary information about the variance and covariance of the estimators can be used to improve the precision of CDF estimators. Let β denotes the ratio of covariance between $\left({\hat{G}}_{\text{S}}^{*}(y),{\hat{G}}_{\text{S}}^{*}(x)\right)$ to the variance of ${\hat{G}}_{\text{S}}^{*}(x)$, under an S sampling scheme, i.e.

$$\beta_{\text{S}}\;=\;\frac{C({\hat{G}}_{\text{S}}^{*}(y),{\hat{G}}_{\text{S}}^{*}(x))}{V({\hat{G}}_{\text{S}}^{*}(x))}.$$ (59)

For situations involving non-response, the difference estimator for the population CDF, say ${\hat{G}}_{D}^{*}(y)$, which relies on $\beta_{\text{S}}$, ${\hat{G}}_{\text{S}}^{*}(y)$, *G*(*x*), and ${\hat{G}}_{\text{S}}^{*}(x)$, is expressed as:

$${\hat{G}}_{D}^{*}(y)\;=\;{\hat{G}}_{\text{S}}^{*}(y)+\beta_{\text{S}}\left(G(x)-{\hat{G}}_{\text{S}}^{*}(x)\right),$$ (60)

where ${\hat{G}}_{D}^{*}(y)$ expressed as a linear aggregator of ${\hat{G}}_{\text{S}}^{*}(y)$ and ${\hat{G}}_{\text{S}}^{*}(x)$, and *G*(*x*) denotes the population CDF under the S sampling scheme. It can be demonstrated that the ${\hat{G}}_{D}^{*}(y)$ is an unbiased estimator of *G*(*y*), and variance of ${\hat{G}}_{D}^{*}(y)$ can be obtained by expressing the Eq (63) in terms of ε’s as:


$${\hat{G}}_{D}^{*}(y)=G(y)(1+\varepsilon_{0})-\beta_{\text{S}}G(x)\varepsilon_{1}$$

$${\hat{G}}_{D}^{*}(y)-G(y)=G(y)\varepsilon_{0}-\beta_{\text{S}}G(x)\varepsilon_{1}.$$ (61)

To calculate the variance of ${\hat{G}}_{D}^{*}(y)$, square both sides of Eq (64) and apply expectation.

$$V({\hat{G}}_{D}^{*}(y))\;=\;G(y{)}^{2}\Lambda_{20}+\beta_{\text{S}}^{2}G(x{)}^{2}\Lambda_{02}-2\beta_{\text{S}}G(x)G(y)\Lambda_{11}.$$ (62)

The simplified equation for the variance of ${\hat{G}}_{D}^{*}(y)$ may be obtained by substituting $\beta_{\text{S}}$, given by

$$V({\hat{G}}_{D}^{*}(y))\;=\;G(y{)}^{2}\Lambda_{20}(1-\kappa^{2}),$$ (63)

which is identical to the minimal MSE of ${\hat{G}}_{R}^{*}(y)$ and ${\hat{G}}_{E}^{*}(y)$.

## 6 Empirical Study

In this section, we consider simulation study and real datasets to calculate the relative efficiencies (REs) of the proposed CDF estimators for *G*(*y*) under non-response, using the S sampling scheme-based estimator ${\hat{G}}_{\text{S}}^{*}(y)$.

### 6.1 Population I

A dataset sourced from the Centers for Disease Control (CDC), is associated with the Second National Health and Nutrition Examination Survey (NHANES-II). It comprises 10,351 units, representing the entire non-institutionalized civilian (NIC) population of the United States (US), including all 50 states and the District of Columbia. The data is separated into four geographic regions (REGs): midwestern, southern, northeastern, and western, each farther subdivided into specific locations (LOCs). With a success probability of 0.50, random numbers are generated from a Bernoulli distribution to stratify the dataset into two strata, with 0 indicating Stratum-I and 1 indicating Stratum-II. The dataset can be found at: https://www.stata-press.com/data/r15/svy.html. Our goal is to determine the percentage of underweight individuals in the NIC US population using body mass index (BMI) as the study variable indices by “Y” and weight as the ancillary variable indices by “X”. An individual is classified as underweight if their BMI is less than or equal to *y* = 18.50, with the average weight of the NIC US population being *x* = 71.8975. For the 2SCS/S2SCS design, REG and BMI are treated as PSU and SSU. In the 3SCS/S3SCS design, REG, LOC, and BMI are considered as PSU, SSU, and TSUs, respectively. Below are the constants for the population.


$$G(y)=0.0318,\beta_{1,X}=0.7364,CV_{X}=0.2136,$$

$$G(x)=0.5401,\beta_{2,X}=4.0614\;\text{and}\;\rho_{XY}=0.8338.$$

To demonstrate what we have aforesaid, the values of $\Lambda_{rs}$ based on an S sampling scheme are computed, which are given below.

**Table 2:** The $\Lambda_{rs}$ values based on an S sampling scheme under non-response utilizing Population I

S	$S_{\text{tr}}-Variable$	PSU	SSU	TSU	$n_{1}$	$n_{2,\text{i}}$	$n_{3,ij}$	$\Lambda_{20}$	$\Lambda_{02}$	$\Lambda_{11}$
2SCS	—-	REG	BMI	—-	3	50	—-	0.31822	0.01009	0.01081
S2SCS	0/1	REG	BMI	—-	3	50	—-	0.15811	0.00503	0.00541
3SCS	—-	REG	LOC	BMI	3	3	50	0.11662	0.00994	0.01055
S3SCS	0/1	REG	LOC	BMI	3	3	50	0.05757	0.00528	0.00538

Note: Stratifying is abbreviated as ${\text{S}}_{tr}$.

### 6.2 Population II

This data is taken from the study conducted by [27] in Multan district, southern Punjab, Pakistan, spans from January 2020 to March 2020. The dataset includes 1040 units, categorized into two strata: Stratum-I (males) and Stratum-II (females). In this study, BMI and weight are used as study and auxiliary variables, respectively. The objective is to estimate the proportion of underweight children by utilizing the auxiliary variable, with *x* = 35.76 representing the average weight, under the S sampling scheme. For the 2SCS/S2SCS design, socioeconomic status (SES) and BMI are treated as PSU and SSU. In the 3SCS/S3SCS design, SES, Age, and BMI are considered as PSU, SSU, and TSUs, respectively. The population constants are as follows:


$$G(y)=0.4741,G(x)=0.5019,CV_{X}=0.3269,$$

$$\rho_{XY}=0.7406,\beta_{1,X}=0.1949\;\text{and}\;\beta_{2,X}=2.6265.$$

To demonstrate what we have aforesaid, the values of $\Lambda_{rs}$ primarily based on an S sampling scheme are computed below.

**Table 3:** The $\Lambda_{rs}$ values based on an S sampling scheme under non-response utilizing Population II

S	$\text{\{\textbackslash{}bf S}{\}}_{\text{tr}}-\text{\{\textbackslash{}bf Variable}\}$	PSU	SSU	TSU	$n_{1}$	$n_{2,i}$	$n_{3,\text{ij}}$	$\Lambda_{20}$	$\Lambda_{02}$	$\Lambda_{11}$
2SCS	—-	SES	BMI	—-	3	50	—-	0.01488	0.01266	0.00858
S2SCS	0/1	SES	BMI	—-	3	50	—-	0.00681	0.00577	0.00396
3SCS	—-	SES	AGE	BMI	3	14	5	0.01369	0.01179	0.00842
S3SCS	0/1	SES	AGE	BMI	3	8	2	0.00609	0.00559	0.00391

We present the following expressions to compute REs of the proposed CDF estimators of finite population distribution function under S sampling schemes with non-response relative to ${\hat{G}}_{\text{S}}^{*}(y)$.


$${\text{RE}}_{R}=\frac{V({\hat{G}}_{\text{S}}^{*}(y))}{\text{MSE}({\hat{G}}_{R}^{*(t)}(y))},\;{\text{RE}}_{E}=\frac{V({\hat{G}}_{\text{S}}^{*}(y))}{\text{MSE}({\hat{G}}_{E}^{*(t)}(y))},\;{\text{RE}}_{D}=\frac{V({\hat{G}}_{\text{S}}^{*}(y))}{V({\hat{G}}_{D}^{*}(y))},$$

where t=1,2,…,9. Table 4 and 5 represent the REs of these CDF estimators.

**Table 4:** Evaluating REs of proposed CDF estimator of *G*(*y*) under non-response relative to ${\hat{G}}_{\text{S}}^{*}(y)$ utilizing Population I

${\hat{G}}_{R}^{*}(y)$	2SCS	S2SCS	3SCS	S3SCS	${\hat{G}}_{E}^{*}(y)$	2SCS	S2SCS	3SCS	S3SCS
${\hat{G}}_{R}^{*(1)}(y)$	1.0376	1.0380	1.1057	1.1054	${\hat{G}}_{E}^{*(1)}(y)$	1.0267	1.0270	1.0743	1.0760
${\hat{G}}_{R}^{*(2)}(y)$	1.0223	1.0225	1.0615	1.0631	${\hat{G}}_{E}^{*(2)}(y)$	1.0123	1.0124	1.0333	1.0344
${\hat{G}}_{R}^{*(3)}(y)$	1.0335	1.0338	1.0939	1.0953	${\hat{G}}_{E}^{*(3)}(y)$	1.0207	1.0209	1.0569	1.0585
${\hat{G}}_{R}^{*(4)}(y)$	1.0076	1.0077	1.0205	1.0211	${\hat{G}}_{E}^{*(4)}(y)$	1.0039	1.0039	1.0104	1.0108
${\hat{G}}_{R}^{*(5)}(y)$	1.0019	1.0019	1.0050	1.0051	${\hat{G}}_{E}^{*(5)}(y)$	1.0009	1.0009	1.0025	1.0026
${\hat{G}}_{R}^{*(6)}(y)$	1.0369	1.0373	1.1038	1.1042	${\hat{G}}_{E}^{*(6)}(y)$	1.0250	1.0252	1.0692	1.0709
${\hat{G}}_{R}^{*(7)}(y)$	1.0325	1.0328	1.0911	1.0925	${\hat{G}}_{E}^{*(7)}(y)$	1.0198	1.0199	1.0543	1.0559
${\hat{G}}_{R}^{*(8)}(y)$	1.0078	1.0079	1.0212	1.0219	${\hat{G}}_{E}^{*(8)}(y)$	1.0040	1.0041	1.0108	1.0112
${\hat{G}}_{R}^{*(9)}(y)$	1.0236	1.0238	1.0653	1.0669	${\hat{G}}_{E}^{*(9)}(y)$	1.0131	1.0132	1.0357	1.0368
${\hat{G}}_{D}^{*}(y)$	1.0377	1.0382	1.1061	1.1055					

**Table 5:** Evaluating REs of the proposed CDF estimator of *G*(*y*) under non-response relative to ${\hat{G}}_{\text{S}}^{*}(y)$ utilizing Population-II

${\hat{G}}_{R}^{*}(y)$	2SCS	S2SCS	3SCS	S3SCS	${\hat{G}}_{E}^{*}(y)$	2SCS	S2SCS	3SCS	S3SCS
${\hat{G}}_{R}^{*(1)}(y)$	1.4345	1.4644	1.5839	1.5704	${\hat{G}}_{E}^{*(1)}(y)$	1.5726	1.5881	1.6655	1.6980
${\hat{G}}_{R}^{*(2)}(y)$	1.4863	1.4971	1.5534	1.5816	${\hat{G}}_{E}^{*(2)}(y)$	1.2473	1.2509	1.2711	1.2842
${\hat{G}}_{R}^{*(3)}(y)$	1.6303	1.6511	1.7511	1.7832	${\hat{G}}_{E}^{*(3)}(y)$	1.3723	1.3789	1.4153	1.4363
${\hat{G}}_{R}^{*(4)}(y)$	1.1950	1.1976	1.2123	1.2224	${\hat{G}}_{E}^{*(4)}(y)$	1.0954	1.0964	1.1027	1.1073
${\hat{G}}_{R}^{*(5)}(y)$	1.0694	1.0701	1.0745	1.0778	${\hat{G}}_{E}^{*(5)}(y)$	1.0343	1.0347	1.0367	1.0383
${\hat{G}}_{R}^{*(6)}(y)$	1.6081	1.6366	1.7621	1.7768	${\hat{G}}_{E}^{*(6)}(y)$	1.4829	1.4935	1.5491	1.5771
${\hat{G}}_{R}^{*(7)}(y)$	1.5948	1.6119	1.6965	1.7295	${\hat{G}}_{E}^{*(7)}(y)$	1.3275	1.3329	1.3628	1.3809
${\hat{G}}_{R}^{*(8)}(y)$	1.2213	1.2244	1.2418	1.2534	${\hat{G}}_{E}^{*(8)}(y)$	1.1082	1.1094	1.1166	1.1219
${\hat{G}}_{R}^{*(9)}(y)$	1.6382	1.6641	1.7826	1.8068	${\hat{G}}_{E}^{*(9)}(y)$	1.4392	1.4480	1.4953	1.5206
${\hat{G}}_{D}^{*}(y)$	1.6423	1.6666	1.7827	1.8083					

Based on the S sampling scheme, the REs of the proposed CDF estimators were estimated with the distinctive values of *n*_1_, *n*_2,*i*_, and *n*_3,*ij*_ using the aforementioned datasets. It can be observed that proposed CDF estimators under S sampling schemes that utilizing supplementary information appear to be marginally more efficient than do not, as indicated by RE values greater than one. Furthermore, CDF estimators for S sampling schemes with stratification can also be seen slightly more efficient than those without stratification. Efficiency tends to increase with the number of sampling stages, although the impact of increasing the sample size remains uncertain. Typically, RE tends to increase by increasing the sample size at various sampling stages and vice varsa.

### 6.3 Simulation Study

To further validate our findings, we estimated the MSE through a simulation approach by computing the squared difference between the estimator and the true parameter. The CDF of the proposed family of estimators under different non-response rates and sample sizes were simulated, and their corresponding MSEs were calculated using a simulation-based approach. The MSE/Variance of the CDF estimators under non-response are computed by drawing 10 thousand samples from Population I under a given sampling scheme. The simulation MSE of estimators for both families under S-sampling scheme are computed as:

$${\text{MSE}}_{\text{Simulated}}({\hat{G}}_{R}^{*(t)}(y))\approx \frac{1}{\theta }\sum_{i=1}^{\theta }({\hat{G}}_{R,\theta }^{*(t)}(y)-F(y){)}^{2}$$ (64)

$${\text{MSE}}_{\text{Simulated}}({\hat{G}}_{E}^{*(t)}(y))\approx \frac{1}{\theta }\sum_{i=1}^{\theta }({\hat{G}}_{E,\theta }^{*(t)}(y)-F(y){)}^{2}$$ (65)

$${\text{Var}}_{\text{Simulated}}({\hat{G}}_{S}^{*}(y))\approx \frac{1}{\theta }\sum_{i=1}^{\theta }({\hat{G}}_{S,\theta }^{*}(y)-F(y){)}^{2}$$ (66)

where θ=10000. The relative precision (RP) of ${\hat{G}}_{R}^{*(t)}(y)$ and ${\hat{G}}_{E}^{*(t)}(y)$ with respect to ${\hat{G}}_{S}^{*}(y)$ under an S-sampling scheme is given by:

$${\text{RP}}_{R}=\frac{{\text{Var}}_{\text{Simulated}}({\hat{G}}_{S}^{*}(y))}{{\text{MSE}}_{\text{Simulated}}({\hat{G}}_{R}^{*(t)}(y))},\;{\text{RP}}_{E}=\frac{{\text{Var}}_{\text{Simulated}}({\hat{G}}_{S}^{*}(y))}{{\text{MSE}}_{\text{Simulated}}({\hat{G}}_{E}^{*(t)}(y))},\;$$ (67)

We then calculated the RP between the estimators and found that our proposed classical ratio/product-type, exponential ratio/product-type, and difference CDF estimators performed well compared to existing methods. Additionally, we extended our simulation analysis by computing the MSE for different sample sizes of PSUs, SSUs, and TSUs under 2SCS/S2SCS, and 3SCS/S3SCS schemes. To assess the robustness of our estimators under varying levels of non-response, we evaluated their efficiency at response rates of 75% and 80%. For brevity of discussion, we considered *c* = 1 in our calculations.

While estimating the MSE through simulation, we adopted this structured sampling design for selecting PSUs, SSUs, and TSUs under different sampling schemes. This framework was applied across 2SCS, 3SCS, and their stratified counterparts, ensuring a systematic and realistic representation of hierarchical data selection and can bee seen in Table 6. This structured approach allowed us to analyze the performance of the proposed estimators under varying levels of non-response effectively.

**Table 6:** The structured sampling design for the estimation of MSE through simulation under non-response utilizing Population I

	$S_{\text{tr}}-\text{Variable}$	PSU	SSU	TSU	*Y*	*X*
2SCS	–	Location	BMI & Weight	–	BMI	Weight
S2SCS	0/1	Location	BMI & Weight	–	BMI	Weight
3SCS	–	Region	Location	BMI & Weight	BMI	Weight
S3SCS	0/1	Region	Location	BMI & Weight	BMI	Weight

Overall, the proposed families of estimators performed well under different sampling conditions. However, the proposed difference estimator demonstrated slightly higher efficiency compared to both the estimators without auxiliary information and the other proposed families of estimators. Although the efficiency gains of the difference estimator were modest, it consistently outperformed other estimators across all sampling schemes. Notably, the maximum gain in precision was found in the ratio/product-type estimators, particularly under the 3SCS scheme, where it reached 15% when considering a sampling structure of *n*_1_ = 2, *n*_2,*i*_ = 12, and *n*_3,*ij*_ = 20.

The consistency between the theoretical and simulation-based MSE results further validates the reliability of the proposed estimators in handling non-response within complex survey sampling frameworks. The detailed results of these simulation studies under various complex survey sampling schemes and non-response rates can be found in the supplementary file (S1 Tables), from Table 1 to Table 16.

## 7 Conclusion

This paper considered 2SCS and 3SCS schemes with and without stratification under non-response in order to estimate the finite population CDF. Two families of estimators, namely exponential and ratio/product-type, have been proposed. Additionally, the CDF has also been estimated using a difference estimator. Under the first order of approximation, mathematical expressions have been developed for the biases and MSE of the prospective CDF estimators under these sampling schemes. Real datasets were used to substantiate and illustrate the proposed theory. The CDF estimators proposed for non-response in complex survey sampling with auxiliary information appeared to be marginally more efficient than those without ancillary details. Our findings revealed that the difference estimators performed well in terms of RE compared to the other estimators discussed in this study. Moreover, the results of the simulation study further confirmed the efficiency of the proposed families of estimators, demonstrating their superior performance across different sampling scenarios. The simulations provided additional evidence that the proposed families of estimators consistently outperformed other estimators, reinforcing our theoretical findings.

**Table 7:** List of abbreviations and acronyms

Acronym	Full Term	Reference
CDF	Cumulative Distribution Function	[20]
2SCS	Two-Stage Cluster Sampling	[4]
3SCS	Two-Stage Cluster Sampling	[4]
S2SCS	Stratified Two-Stage Cluster Sampling	[19]
S3SCS	Stratified Three-Stage Cluster Sampling	[1]
SRS	Simple Random Sampling	[4]
2SCS-SRS	2SCS with SRS in Second-Stage of Sampling	[10]
S2SCS-SRS	S2SCS with SRS in Second-Stage of Sampling	[10]
3SCS-SRS	3SCS with SRS in Third-Stage of Sampling	[10]
S3SCS-SRS	S3SCS with SRS in Second-Stage of Sampling	[10]
PSUs	Primary Sampling Units	[4]
SSUs	Secondary Sampling Units	[4]
TSUs	Tertiary Sampling Units	[4]
REs	Relative Efficiencies	[4]
RPs	Relative Precision	[4]
BMI	Body Mass Index	[10]
SES	Socioeconomic Status	[27]

## Supporting information

S1 Tables Simulation Results of the RP of the Proposed CDF Estimators. This file contains RP of the proposed CDF estimators under non-response using auxiliary information within 2SCS/3SCS, and S2SCS/S3SCS schemes
(TEX)

S2 Files Datasets for Population I, and II. This file contains datasets supporting the findings of this study.
(XLSX)

S3 Appendix Proofs for Lemma 7 and 9. This file contains demonstration of Lemma 7 and Lemma 9.
(PDF)
