# Supplementary material for: Estimation of the distribution function of a finite population utilizing auxiliary information in the context of non-response within complex survey sampling
Source: PLoS One. 2025 May 22;20(5):e0322660. doi: 10.1371/journal.pone.0322660 (PMC12097595; doi:10.1371/journal.pone.0322660)
Supplement: S3 Appendix — This file contains demonstration of Lemma 7 and Lemma 9. (PDF) [file pone.0322660.s003.pdf]

Appendix

This Appendix contains the detailed substantiation of Lemmas discussed in Section 4: Covariance computation and estimation in the presence of non-response.

1: Substantiation of Lemma 7

By assigning basis "1", and "2" to represent the first, and second stages of sampling, separately, we can express the covariance between $\hat{G}^*_{2\mathrm{S}}(y)$ and $\hat{G}^*_{2\mathrm{S}}(x)$ as:

$$C(\hat{G}^*_{2\mathrm{S}}(y), \hat{G}^*_{2\mathrm{S}}(x)) \;=\; C_1[E_2(\hat{G}^*_{2\mathrm{S}}(y), \hat{G}^*_{2\mathrm{S}}(x))] + E_1[C_2(\hat{G}^*_{2\mathrm{S}}(y), \hat{G}^*_{2\mathrm{S}}(x))]. \tag{1}$$

It can be demonstrated that $E_2(\hat{G}^*_{2\mathrm{S}}(y)) = \sum_{i=1}^{n} N_{2,i}G_i(y)/(n\overline{N})$. Based on this outcome, we have

$$\begin{aligned}
C_1\left[E_2(\hat{G}^*_{2\mathrm{S}}(y), \hat{G}^*_{2\mathrm{S}}(x))\right] &= C_1\left(\frac{1}{n_1\overline{N}}\sum_{i=1}^{n_1} N_{2,i}G_i(y), \frac{1}{n_1\overline{N}}\sum_{i=1}^{n_1} N_{2,i}G_i(x)\right)\\
&= \frac{\lambda\sigma_{XY,2,b}}{n_1\overline{N}^2}, \qquad (2)\\
E_1\left[C_2(\hat{G}^*_{2\mathrm{S}}(y), \hat{G}^*_{2\mathrm{S}}(x))\right] &= E_1\left[\frac{1}{n_1^2\overline{N}^2}\sum_{i=1}^{n_1} N_{2,i}^2\, C_2(\hat{G}_i(y), \hat{G}_i(x))\right],\\
&= \frac{1}{n_1N_1}\sum_{i=1}^{N_1}\frac{N_{2,i}^2}{\overline{N}^2}\left[\frac{\lambda_i\sigma_{XY,2,i}}{n_{2,i}} + \frac{W_i^*(c-1)\sigma^*_{XY,2,i}}{n_{2,i}}\right] \qquad (3)
\end{aligned}$$

which completes the proof.

2: Substantiation of Lemma 9

By assigning basis "1", "2", and "3" to represent the first, second, and third stages of sampling, separately, we can express the covariance between $\hat{G}^*_{3\mathrm{S}}(y)$ and $\hat{G}^*_{3\mathrm{S}}(x)$ as:

$$\begin{aligned}
C\left(\hat{G}^*_{3\mathrm{S}}(y), \hat{G}^*_{3\mathrm{S}}(x)\right) &= C_1E_2E_3\left[\hat{G}^*_{3\mathrm{S}}(y), \hat{G}^*_{3\mathrm{S}}(x)\right] + E_1C_2E_3\left[\hat{G}^*_{3\mathrm{S}}(y), \hat{G}^*_{3\mathrm{S}}(x)\right]\\
&\quad + E_1E_2C_3\left[\hat{G}^*_{3\mathrm{S}}(y), \hat{G}^*_{3\mathrm{S}}(x)\right]. \qquad (4)
\end{aligned}$$

From Eq. (**??**), $E_3(\hat{G}^*_{3\mathrm{S}}(y)) = \sum_{i=1}^{n_1}(N_{2,i}/n_{2,i})\sum_{j=1}^{n_{2,i}} N_{3,ij}G_{ij}(y)/n_1\overline{T}$. Based on this result, we have

$$\begin{aligned}
C_1E_2E_3\left[\hat{G}^*_{3\mathrm{S}}(y), \hat{G}^*_{3\mathrm{S}}(x)\right] &= C_1E_2\left[\frac{1}{n_1\overline{T}}\sum_{i=1}^{n_1}\frac{N_{2,i}}{n_{2,i}}\sum_{j=1}^{n_{2,i}} N_{3,ij}G_{ij}(y),\right.\\
&\qquad \left.\frac{1}{n_1\overline{T}}\sum_{i=1}^{n_1}\frac{N_{2,i}}{n_{2,i}}\sum_{j=1}^{n_{2,i}} N_{3,ij}G_{ij}(x)\right]\\
&= C_1\left[\frac{1}{n_1\overline{T}}\sum_{i=1}^{n_1} N_{2,i}G_i(y), \frac{1}{n_1\overline{T}}\sum_{i=1}^{n_1} N_{2,i}G_i(x)\right]\\
&= \frac{\lambda\sigma_{XY,3,b}}{n_1\overline{T}^2}. \qquad (5)
\end{aligned}$$

$$
\begin{aligned}
E_1C_2E_3\left[\hat{G}^*_{3\mathrm{S}}(y),\hat{G}^*_{3\mathrm{S}}(x)\right] &= E_1C_2\left[\frac{1}{n_1\overline{T}}\sum_{i=1}^{n_1}\frac{N_{2,i}}{n_{2,i}}\sum_{j=1}^{n_{2,i}}N_{3,ij}G_{ij}(y),\right.\\
&\left.\frac{1}{n_1\overline{T}}\sum_{i=1}^{n_1}\frac{N_{2,i}}{n_{2,i}}\sum_{j=1}^{n_{2,i}}N_{3,ij}G_{ij}(x)\right]\\
&= E_1\left[\frac{1}{n_1^2\overline{T}^2}\sum_{i=1}^{n_1}N_{2,i}^2\ C_2\Big(\frac{1}{n_{2,i}}\sum_{j=1}^{n_{2,i}}N_{3,ij}G_{ij}(y),\right.\\
&\left.\frac{1}{n_{2,i}}\sum_{j=1}^{n_{2,i}}N_{3,ij}G_{ij}(x)\Big)\right]\\
&= E_1\left[\frac{1}{n_1^2\overline{T}^2}\sum_{i=1}^{n_1}\frac{\lambda_iN_{2,i}^2\sigma_{XY,3,i}}{n_{2,i}}\right]\\
&= \frac{1}{n_1N_1\overline{T}^2}\sum_{i=1}^{N_1}\frac{\lambda_iN_{2,i}^2\sigma_{XY,3,i}}{n_{2,i}}. \qquad (6)
\end{aligned}
$$

$$
\begin{aligned}
E_1E_2C_3\left[\hat{G}^*_{3\mathrm{S}}(y),\hat{G}^*_{3\mathrm{S}}(x)\right] &= E_1E_2\left[\frac{1}{n_1^2\overline{T}^2}\sum_{i=1}^{n_1}\frac{N_{2,i}^2}{n_{2,i}^2}\sum_{j=1}^{n_{2,i}}N_{3,ij}^2\ C_3\big(\hat{G}_{ij}(y),\hat{G}_{ij}(x)\big)\right]\\
&= E_1E_2\left[\frac{1}{n_1^2}\sum_{i=1}^{n_1}\frac{N_{2,i}^2}{n_{2,i}^2}\sum_{j=1}^{n_{2,i}}\frac{N_{3,ij}^2}{\overline{T}^2}\Big(\frac{\lambda_{ij}\sigma_{XY,3ij}}{n_{3,ij}}\right.\\
&\left.+\frac{W^*_{ij}(c-1)\sigma^*_{XY,3,ij}}{n_{3,ij}}\Big)\right]\\
&= E_1\left[\frac{1}{n_1^2}\sum_{i=1}^{n_1}\frac{N_{2,i}}{n_{2,i}}\sum_{j=1}^{N_{2,i}}\frac{N_{3,ij}^2}{\overline{T}^2}\Big(\frac{\lambda_{ij}\sigma_{XY,3,ij}}{n_{3,ij}}+\frac{W^*_{ij}(c-1)\sigma^*_{XY,3,ij}}{n_{3,ij}}\Big)\right]\\
&= \frac{1}{n_1N_1}\sum_{i=1}^{N_1}\frac{N_{2,i}}{n_{2,i}}\sum_{j=1}^{N_{2,i}}\frac{N_{3,ij}^2}{\overline{T}^2}\Big(\frac{\lambda_{ij}\sigma_{XY,3,ij}}{n_{3,ij}}+\frac{W^*_{ij}(c-1)\sigma^*_{XY,3,ij}}{n_{3,ij}}\Big). \qquad (7)
\end{aligned}
$$

Add Eqs. (5)–(7), which completes the proof.
